# Association between the uric acid to high density lipoprotein cholesterol ratio and alanine transaminase in Chinese short stature children and adolescents: A cross-sectional study

**DOI:** 10.3389/fnut.2023.1063534

**Published:** 2023-01-24

**Authors:** Guangxin Li, Qianqian Zhao, Xinran Zhang, Bo Ban, Mei Zhang

**Affiliations:** ^1^Department of Clinical Medicine, Jining Medical University, Jining, Shandong, China; ^2^Department of Endocrinology, Affiliated Hospital of Jining Medical University, Jining Medical University, Jining, Shandong, China; ^3^Chinese Research Center for Behavior Medicine in Growth and Development, Jining, Shandong, China

**Keywords:** uric acid, high density lipoprotein cholesterol, alanine transaminase, short stature, children and adolescents

## Abstract

**Objective:**

Uric acid to high-density lipoprotein cholesterol ratio (UHR), the ratio of uric acid to high-density lipoprotein cholesterol, is a newly proposed marker of metabolic abnormalities. There are few previous studies directly investigating the relationship between UHR and alanine aminotransferase (ALT), especially in short stature populations, however, short stature children and adolescents are more likely to have metabolic disorders. This research aimed to investigate the relationship between the UHR and ALT in children and adolescents with short stature.

**Methods:**

In this cross-sectional analysis, the clinical data of 1,510 children with height below −2 SD who were evaluated at the Department of Endocrinology, Affiliated Hospital of Jining Medical University from 1 March 2013 to 31 December 2021, were selected. Anthropometric and biochemical indicators were measured. The relationship between UHR and ALT was analysed.

**Results:**

The univariate analysis results showed that UHR was positively associated with ALT (β = 0.43, *P* < 0.0001). Furthermore, after adjusting for possible confounding factors, a non-linear relationship was detected between UHR and ALT through smooth curve fitting, and the inflection point of UHR was 10.93% after multivariate piecewise linear regression analysis. ALT increased with UHR elevation when the UHR was greater than 10.93% (β = 0.69, 95% CI 0.39, 0.98; *P* < 0.0001). However, we did not observe a significant relationship when the UHR was less than 10.93% (*P* = 0.9229).

**Conclusion:**

Our study demonstrated that in Chinese children and adolescents with short stature, UHR may be associated with the regulation of ALT levels, and this relationship merits further investigation.

## Introduction

Short stature is defined as a height of less than −2 standard deviations (SDs) compared to the mean height for the corresponding age and sex (1). The most common reasons for short stature are growth hormone deficiency (GHD) and idiopathic short stature (ISS) (2). Growth hormone (GH) is an anterior pituitary hormone, a key regulator of adipolysis that acts on the liver through the GH/insulin-like growth factor-1 (GH/IGF-1) axis (3). Animal experiments have shown that the loss of GH signaling in hepatocytes leads to steatosis and liver damage, promoting the development of metabolic-associated fatty liver disease (MAFLD) (4). MAFLD is a new concept proposed by the International Expert Consensus Group in 2020, and similar to the previous term non-alcoholic fatty liver disease (NAFLD), MAFLD represents the liver manifestations of a multisystem disease (5). With the effective extension of the new definition of MAFLD in adults, it was introduced to children and adolescents in 2021 (6). The increasing global obesity in children and adolescents has prompted an increase in childhood MAFLD. MAFLD is now considered the most common cause of chronic liver disease in adults and children worldwide (7).

A large number of clinical studies suggest that childhood and adolescent GH deficiency are associated with an increased risk of MAFLD (8, 9). By definition, ISS is defined as short stature in childhood with no known aetiology, and although there is no clear cause, some researchers have attempted to explain the underlying mechanism of impaired linear growth in children with ISS (4, 10). The GH/IGF-1 axis plays an important role during the critical period of childhood growth and development. A cross-sectional study in the US showed that reduced serum IGF-1 levels were associated with increased histological severity of MAFLD (11).

MAFLD is assessed in children and adults with serum alanine aminotransferase (ALT) and ultrasound screening, with liver biopsy for diagnosis. In clinical studies, the most commonly used predictor of childhood MAFLD is elevated ALT levels. The MAFLD Expert Committee has advocated the use of ALT as a screening test for children with MAFLD (12). In the absence of any other liver injury, high ALT levels often reflect the presence of MAFLD (13). Therefore, it is necessary to investigate ALT levels and their related factors in short stature children and adolescents.

Children and adolescents with short stature not only have height problems but also, more importantly, have abnormal metabolic function (14). Existing studies have shown that children with short stature have higher odds of developing metabolic syndrome compared to the normal height group, which leads to a greatly increased chance of cardiovascular events in the future (15). Recently, it has been reported that the uric acid to high density lipoprotein cholesterol ratio (UHR) is a new inflammatory and metabolic indicator. It has high sensitivity and specificity as compared to other diagnostic criteria for metabolic syndrome (16). As a novel marker, UHR has been shown to be higher in the MAFLD population (17). In other words, UHR may be related to ALT, but there are few studies in this area, especially in short children and adolescents. Therefore, we conducted this study to explore the relationship between UHR and ALT levels in children and adolescents with short stature.

## Materials and methods

### Study subjects

According to the inclusion and exclusion criteria, the subjects included a total of 1,510 children and adolescents with short stature who visited at the Department of Endocrinology, Affiliated Hospital of Jining Medical University from 1 March 2013 to 31 December 2021. Collect and organize clinical data that meet the diagnostic criteria of short stature, and conduct cross-sectional analysis. Data of this study are part of the cohort GDDSD study (Growth and Development Diseases in Shandong Province: a cohort follow-up study^1^). Subjects were included and excluded according to the following criteria: Inclusion criteria: The height of each subject is less than −2 SDs compared with the same age, same sex, same ethnic group. Exclusion criteria: patients with abnormal thyroid function, small for gestational age, intracranial tumor, Turner syndrome, Noonan syndrome, Kallman syndrome, congenital heart disease, skeletal dysplasia, received GH therapy, abnormal liver function, and subjects with incomplete data on ALT, UA and HDL (Table 1).

**TABLE 1:** Study population description.

All	
Number	1510
Male (%)	1029 (68.2)
Age (years)	10.4 ± 3.6
Height (cm)	125.9 ± 18.0
Height SDS	−2.5 (−3.0, −2.2)
Weight (kg)	27.9 ± 11.3
BMI (kg/m2)	16.9 ± 3.1
SBP (mmHg)	105.8 ± 12.2
DBP (mmHg)	62.6 ± 8.7
IGF-1 (ng/ml)	171.0 (102.0,258.0)
GH peak (ng/ml)	7.0 (4.5,10.5)
HDL-C (mg/dl)	53.6 (46.8,61.2)
LDL-C (mg/dl)	82.0 ± 22.8
TC (mg/dl)	150.3 ± 28.3
TG (mg/dl)	58.5 (46.1,77.5)
ALT (U/L)	15.5 ± 9.0
FBG (mg/dl)	86.5 ± 33.5
UA (mg/dl)	4.5 ± 1.2
CR (μmol/L)	40.8 ± 13.8
UHR (%)	8.7 ± 3.3
**Pubertal stage**	
Prepubertal (%)	936 (62.0)
Pubertal (%)	574 (38.0)

Height SDS, height standard deviation scores; BMI, body mass index; SBP, systolic blood pressure; DBP, diastolic blood pressure; IGF-1, insulin-like growth factor-1; GH peak, growth hormone peak; HDL-C, high density lipoprotein cholesterol; LDL-C, low density lipoprotein cholesterol; TC, total cholesterol; TG, triglyceride; ALT, alanine aminotransferase; FPG, fasting plasma glucose; UA, uric acid; Cr, creatinine; UHR, uric acid to high density lipoprotein cholesterol ratio. Normally distributed data are presented as the mean ± standard deviation; non-normal distributed data are presented as median (interquartile range) and categorical data are presented using number (percentage). *P* < 0.05 was considered to be statistically significant.

### Anthropomorphic measurements

Height and weight of all subjects were measured by trained professionals. When measuring the height, all participants used the same height measuring instrument (Jiangsu Nantong Best Industrial Co., Ltd., China) to measure with an accuracy of 0.1 cm after taking off their hats and shoes. Height SDS was calculated based on normal values for Chinese children (18). When measuring the body weight, all participants were on an empty stomach and wearing light clothes, using the same electronic scale (Guangdong Xiangshan Weighing Apparatus Co., Ltd., China), accurate to 0.1 kg. BMI is equal to weight (kg)/height (meter squared). The division of puberty stages is assessed by a specialist physician through a physical examination, which is based on the Turner stage (19). Boys with testicular volume less than 4 mL and no pubic hair and girls with no breast development and no pubic hair were classified as prepubertal (20, 21). Measurement of systolic blood pressure (SBP) and diastolic blood pressure (DBP) requires the patient to sit and rest for at least 5 min, skilled nurses use Omron HBP-1300 electronic sphygmomanometer to measure the blood pressure of the right arm three times, and the interval between each measurement is not less than 2 min. Then, the average of the SBP and DBP measurements was calculated and recorded.

### Laboratory measurements

Morning fasting blood samples were collected from all patients to determine laboratory parameters. Two types of GH stimulation tests are required to determine GH peak. The first trial was the levodopa excitation test. The specific methods are as follows: Participants weighing < 30 kg received oral levodopa 0.25 g, participants weighing ≥ 30 kg received oral levodopa 0.5 g, blood samples were collected at 0, 30, 60, 90, and 120 min and GH concentrations were determined. The second trial was the insulin hypoglycemia test. The specific method is as follows: 0.1 U/kg insulin was injected subcutaneously, and GH levels were measured at time points of 0, 15, 30, 60, 90, and 120 min, respectively. The GH concentration was determined by a chemiluminescence method (ACCESS2, Beckman Coulter; USA) with a sensitivity of 0.010 μg/l. Serum IGF-1 concentration was determined by the chemiluminescence immunometric method (DPC IMMULITE 1000 analyser, SIEMENS, Germany), and the intra-assay and inter-assay coefficients were 3.0 and 6.2%, respectively. Renal function-related indicators including creatinine (Cr) and uric acid (UA), blood lipid-related indicators including total cholesterol (TC), high-density lipoprotein cholesterol (HDL-c), low-density lipoprotein C (LDL-c), and triglycerides (TG), with fasting blood glucose (FBG), alanine aminotransferase (ALT) were measured by an automatic biochemical analyzer (Cobas c702, Roche; Shanghai, China). The UHR (%) was obtained as UA (mg/dl)/HDL (mg/dl)X100.

### Statistical analysis

Continuous variables that fit the normal distribution are expressed as mean ± standard deviation; if not, the median (interquartile range) is used. First, univariate analysis was used to determine the association between UHR and ALT and other independent variables. The relationship between UHR and ALT was then investigated using smooth curve fitting after adjusting for potential confounders. Finally, a multivariate piecewise linear regression model was applied to test the threshold association between UHR and ALT. Further divide the UHR value into quartiles, and analyze the association of UHR quartiles with the prevalence of elevated ALT was analyzed by multiple regression. *P*-values < 0.05 (two-sided) were considered statistically significant. All data were analyzed using the statistical packages R (^2^ The R Foundation) and EmpowerStats (^3^ X&Y Solutions, Inc., Boston, MA, USA) conduct.

## Results

### Subject population description

Table 1 describes the clinical characteristics of the participants. A total of 1,029 males and 481 females, aged 10.38 ± 3.58 years were enrolled in this study; the mean UHR was 8.73 ± 3.31%, and the mean ALT was 15.51 ± 9.04 U/L.

### Factors associated with ALT

As shown in Table 2, univariate linear regression analysis was performed to determine the relationships between the clinical parameters and ALT. For the unadjusted model, UHR and ALT showed a significant positive correlation (*p* < 0.001). Other variables significantly associated with ALT were sex, age, height SDS, weight, BMI, SBP, DBP, UA, TG, GH peak, and puberty stage (*P* < 0.05). There was no significant correlation between serum ALT and IGF-1, FBG, Cr, HDL, LDL, or TC (*p* > 0.05).

**TABLE 2:** Factors correlated with ALT (U/L) in the subjects.

Variables	β	(95% CI)	*P* value
Age	0.38	(0.26, 0.51)	<0.0001
Height SDS	-0.69	(−1.27, 0.15)	0.0170
Weight	0.15	(0.12, 0.19)	<0.0001
BMI	0.61	(0.47, 0.76)	<0.0001
SBP	0.10	(0.06, 0.13)	<0.0001
DBP	0.11	(0.06, 0.16)	<0.0001
IGF-1	0.00	(−0.00, 0.01)	0.1243
GH peak	-0.13	(−0.21, −0.05)	0.0020
FBG	0.00	(−0.01, 0.02)	0.7057
UA	0.91	(0.54, 1.29)	<0.0001
CR	0.01	(−0.02, 0.04)	0.6138
HDL-C	-0.02	(−0.05, 0.02)	0.4045
LDL-C	0.02	(−0.00, 0.04)	0.1063
TC	0.01	(−0.00, 0.03)	0.1412
TG	0.03	(0.01, 0.04)	<0.0001
UHR	0.43	(0.29, 0.56)	<0.0001
**Sex**			
Male	Reference		
Female	-1.27	(−2.25, −0.29)	0.0111
**Pubertal stage**			
Prepubertal	Reference		
Pubertal	2.00	(1.07, 2.94)	<0.0001

CI, confidence interval; Height SDS, height standard deviation scores; BMI, body mass index; SBP, systolic blood pressure; DBP, diastolic blood pressure; IGF-1, insulin-like growth factor-1; GH peak, growth hormone peak; FPG, fasting plasma glucose; UA, uric acid; Cr, creatinine; HDL-C, high-density lipoprotein cholesterol; LDL-C, low-density lipoprotein cholesterol; TC, total cholesterol; TG, triglyceride; UHR, uric acid to high-density lipoprotein cholesterol ratio. *P* < 0.05 was considered to be statistically significant.

### Independent correlation between UHR and ALT by multivariate piecewise linear regression

**FIGURE 1:**
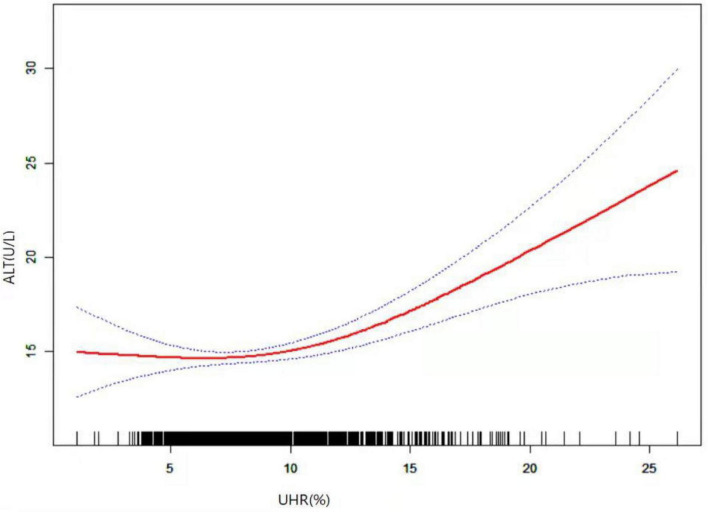
Relationship between UHR and ALT by smooth curve fitting. Adjustment variables: sex, age, puberty stage, BMI, SBP, DBP, and GH peak. BMI, body mass index; SBP, systolic blood pressure; DBP, diastolic blood pressure; GH peak, growth hormone peak; UHR, uric acid to high density lipoprotein cholesterol ratio; ALT, alanine aminotransferase.

As shown in Figure 1, smooth curve fitting revealed a non-linear relationship between UHR and ALT. After adjusting for sex, age, BMI, SBP, DBP, GH peak, and Tanner staging, smooth curve fitting showed a non-linear relationship between UHR and ALT. This curve has two phase changes and a breakpoint. When the UHR levels were less than the critical point, UHR was inversely associated with ALT. When the UHR levels were greater than the critical point, the UHR was positively associated with ALT. As shown in Table 3, the threshold effects were further analysed by curve fitting, and the data indicated that the inflection point of UHR was 10.93%. When UHR was > 10.93%, ALT levels significantly increased with increasing UHR (0.69, 95% CI 0.39, 0.98; *P* < 0.0001). When UHR was < 10.93%, the ALT levels decreased with increasing UHR (−0.01, 95% CI −0.24, 0.21; *P* = 0.9229). However, it was not statistically significant.

**TABLE 3:** Independent correlation between UHR and ALT by multivariate piecewise linear regression.

Inflection point of UHR (%)	Effect size (β)	95% CI	*P* value
<10.93	−0.01	(−0.24, 0.21)	0.9229
≥10.93	0.69	(0.39, 0.98)	<0.0001

Adjustment variables: sex, age, puberty stage, BMI, SBP, DBP, and GH peak. BMI, body mass index; SBP, systolic blood pressure; DBP, diastolic blood pressure; GH: peak, growth hormone peak; UHR, uric acid to high density lipoprotein cholesterol ratio. *p* < 0.05 was considered to be statistically significant.

### The association of UHR quartiles with the prevalence of elevated ALT was analyzed by multiple regression

According to relevant literature reports (22), ALT level rise is defined as ALT ≥ 30 U/L. There are 52 people in our study population with high ALT level. In order to further explore the relationship between UHR and ALT, we divided all participants into quartiles according to their UHR values, as follows: Quartile 1: UHR ≤ 6.39%; Quartile 2: 6.39% < UHR ≤ 8.14%; Quartile 3: 8.14% < UHR ≤ 10.37%; Quartile 4: UHR > 10.37%. And analyzed the association between the quartiles of UHR and the prevalence of elevated ALT through multiple regression analysis (Table 4). We found a positive correlation between the UHR quartile and the prevalence of elevated ALT, which was 1.51% in the first UHR quintile and increased to 1.81, 3.62, and 8.73% in the second, third, and fourth quintile groups, respectively. In addition, we found a significant positive correlation between the fourth UHR quartile and the prevalence of elevated ALT (*p* < 0.001).

**TABLE 4:** Association between UHR quartiles and the prevalence of elevated ALT in patients.

UHR quartile	Total	Elevated ALT	PR%	β (95% CI)	*P* value
Quartile 1	331	5	1.51	0.7 (0.4, 1.5)	0.394
Quartile 2	331	6	1.81	1.2 (0.2, 7.9)	0.823
Quartile 3	331	12	3.62	1.6 (0.6, 4.2)	0.354
Quartile 4	332	29	8.73	1.3 (1.1, 1.5)	< 0.001

Adjustment variables: sex, age, puberty stage, BMI, SBP, DBP, and GH peak. BMI, body mass index; SBP, systolic blood pressure; DBP, diastolic blood pressure; GH: peak, growth hormone peak; UHR, uric acid to high density lipoprotein cholesterol ratio. *p* < 0.05 was considered to be statistically significant.

### Subgroup analysis to assess the relationship between UHR and ALT by smoothing curve fitting

Stratified analysis was performed according to gender, GH peak level, pubertal stage, and the results are in the additional file: (Supplementary Figures 1–3). The association between UHR and ALT was consistent in the subgroup analysis.

## Discussion

In this cross-sectional study, we observed a non-linear relationship between UHR and ALT in short stature children and adolescents, with a turning point of ALT of 10.93%. When UHR levels were greater than 10.93%, UHR was positively associated with ALT.

We observed a positive correlation between UHR and ALT. Previous studies that have directly explored UHR and ALT are limited, but UHR is a new metabolic predictor with high sensitivity and specificity compared to other metabolic syndrome diagnostic criteria (15). Regarding the association between UHR and ALT, Mehmet Ali Kosekli et al. observed that UHR was positively associated with ALT through a controlled study (17). Moreover, a positive association between UHR and ALT was also reported in Chinese lean individuals by Ya-Nan Zhang et al. (23). These results are consistent with the findings of our study. Importantly, these studies only explored the linear relationship between UHR and ALT, and did not further study the non-linear relationship between UHR and ALT. We used a multivariate piecewise linear regression model to test the threshold correlation between UHR and ALT. As for why we draw different conclusion, it may be due to different population characteristics, sample size and adjustment confounding factors. Our results show that there is a strong correlation between UHR and ALT only when UHR is above a certain level, and a simple linear assessment may underestimate this correlation. In addition, the cut-off value of UHR obtained by us is close to the level of UHR reported in previous studies evaluating MAFLD. Previous studies have shown by ROC analysis that a UHR equal to 9.6% is the threshold for determining MAFLD (17). However, the threshold value of UHR obtained in our study on the relationship between UHR and ALT is 10.93%. When the UHR is greater than 10.93%, the ALT level of children and adolescents with short stature increases with the increase of UHR. This finding provides some research basis for the standardized treatment and early intervention of patients with short stature.

Uric acid is a product of purine metabolism, and elevated serum uric acid levels are associated with the deterioration of metabolic status (24). Low HDL-C is also associated with a poor metabolic status and is even a marker of metabolic syndrome (25). The combination of these two metabolic parameters is UHR, a more useful predictor of metabolic deterioration (26). Since hepatic steatosis is associated with metabolic syndrome (27, 28), and ALT reflects an excessive deposition of hepatic fat (29), this could explain the increase in ALT with increased UHR.

Uric acid is synthesized by xanthine oxidase during purine metabolism and is excreted through the kidney. Thus, elevated uric acid levels are a result of increased synthesis and decreased excretion (16, 30). Elevated uric acid levels contribute to the development of adverse conditions. Previous results suggest a strong association between uric acid levels and metabolic syndrome in children and adolescents, and various mechanisms have been proposed to explain this correlation (31). HDL-C has the effects of reverse transport of cholesterol to reduce atherosclerosis, anti-inflammation, anti-thrombosis, antiapoptosis, and vasodilation (32). An analysis of a large health and nutrition survey from the United States indicated that low levels of serum HDL are important in the development of metabolic syndrome and identified it as the most powerful predictor (33). Thus, low serum HDL-C levels with high UA levels reflect a worse metabolic status.

The liver is an important organ of human metabolism. Aging of the liver and abnormal accumulation of fat can rupture hepatocytes, releasing ALT into the blood. Measurement of ALT levels is a basic test for screening for liver disease and assessing disease progression. However, serum ALT is not only a sensitive indicator for assessing liver function but is also closely related to metabolic factors. In China, through a 7-year follow-up cohort study, even if ALT remained within the range of normal reference values, it was an independent predictor of metabolic syndrome (34). In other words, both UHR and ALT are associated with metabolic syndrome, and interestingly, our study found a non-linear relationship between UHR and ALT, with ALT levels increasing with UHR when UHR was above 10.93%, while at lower values, no relationship was observed. It is well known that obesity is a major risk factor for metabolic deterioration. BMI is a useful indicator to assess obesity and nutritional status (35). We found a close relationship between ALT and BMI. However, after adjustment for BMI, the UHR remained independently associated with ALT.

Furthermore, UHR, as a novel marker of metabolic syndrome (36), has been used to assess type 2 diabetes mellitus (16, 26), hepatic steatosis (37), Hashimoto’s thyroiditis (38), and the control of MAFLD (17). UHR was also reported to be associated with poor collateral circulation in chronic total occlusion (CTO) patients and to have important predictive value for cardiovascular mortality in patients living on peritoneal dialysis (39, 40). Evidence has shown that short stature children and adolescents also have metabolic abnormalities, so it is necessary to evaluate UHR levels in the diagnosis and treatment of short stature (14).

## Study strengths and limitations

The present study has several advantages. First, UHR is a newly proposed novel predictor of metabolism, with previous studies on UHR being few and all in adults; this is the first study of the relationship between UHR and ALT levels in short children and adolescents. Second, in this study, we used smooth curve fitting to find a non-linear relationship between UHR and ALT levels rather than assuming a simple positive correlation.

However, this study has some limitations. First, we were unable to determine the causal relationships due to the cross-sectional study design. Second, this study was conducted in a homogeneous population of short-stature children and adolescents in China, so our results cannot be extrapolated to other groups. Third, serum ALT is used as an independent variable to screen MAFLD, but imaging examination is often used to diagnose MAFLD in clinic. In fact, in our study, abdominal ultrasound was used to diagnose MAFLD, but MAFLD was not found. It may be that ultrasound is not ideal for detecting the early stage of MAFLD, and more accurate methods should be used in the future to assess the histological severity of the liver. Finally, according to the diagnostic criteria of metabolic syndrome (37), only seven people in our study population meet this diagnosis, and the prevalence rate of metabolic syndrome is only 0.46%, which limits the description of whether the study population is suffering from metabolic syndrome. In the future, we need to expand the sample size to carry out this study.

## Conclusion

This study describes a non-linear relationship between UHR and ALT levels in Chinese short stature children and adolescents. When the UHR values reached the inflection point, the ALT levels were positively correlated with elevated UHR. This finding suggests that UHR in short stature children and adolescents affects ALT concentrations, so we should focus on the level of uric acid and HDL clinically.

## Data availability statement

The original contributions presented in this study are included in the article/Supplementary material, further inquiries can be directed to the corresponding authors.

## Ethics statement

The studies involving human participants were reviewed and approved by the Human Ethics Committee of the Affiliated Hospital of Jining Medical University. Written informed consent to participate in this study was provided by the participants’ legal guardian/next of kin.

## Author contributions

GL carried out the studies and drafted the manuscript. QZ and XZ helped with the statistical analysis. MZ and BB participated in the conceptualization and design of the study, revised the manuscript critically for important intellectual content, and final provided approval of the version to be published. All authors read and approved the final manuscript.

## Footnotes

1 http://www.chictr.org.cn, ChiCTR1900026510

2
http://www.R-project.org

3
http://www.empowerstats.com
