# Supplementary material for: Association between the uric acid to high density lipoprotein cholesterol ratio and alanine transaminase in Chinese short stature children and adolescents: A cross-sectional study
Source: Front Nutr. 2023 Jan 24;10:1063534. doi: 10.3389/fnut.2023.1063534 (PMC9902588; doi:10.3389/fnut.2023.1063534)
Supplement: Supplementary file 1 [file Data_Sheet_1.pdf]

Additional file:

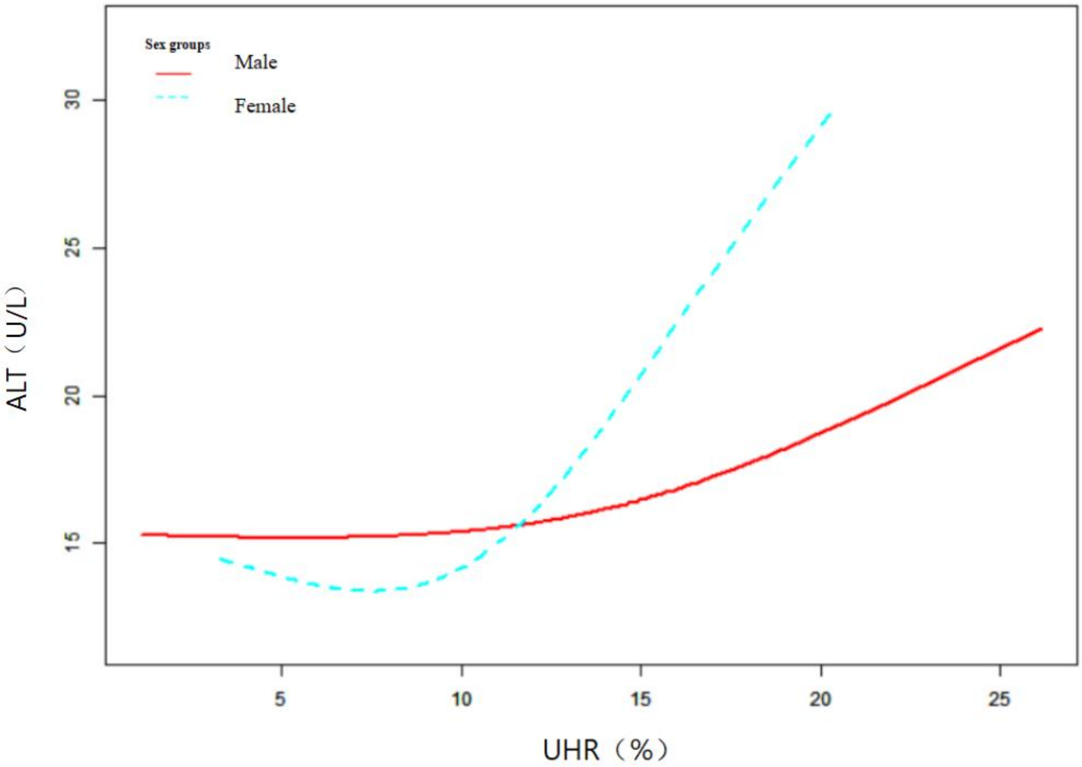

Figure S1: *Smooth curve fitting for the relationship between UHR and ALT stratified by sex groups*

Adjustment variables: age, puberty stage, BMI, SBP, DBP, and GH peak. BMI: body mass index; SBP: systolic blood pressure; DBP: diastolic blood pressure; GH: peak: growth hormone peak; UHR: uric acid to high density lipoprotein cholesterol ratio. p<0.05 was considered to be statistically significant.

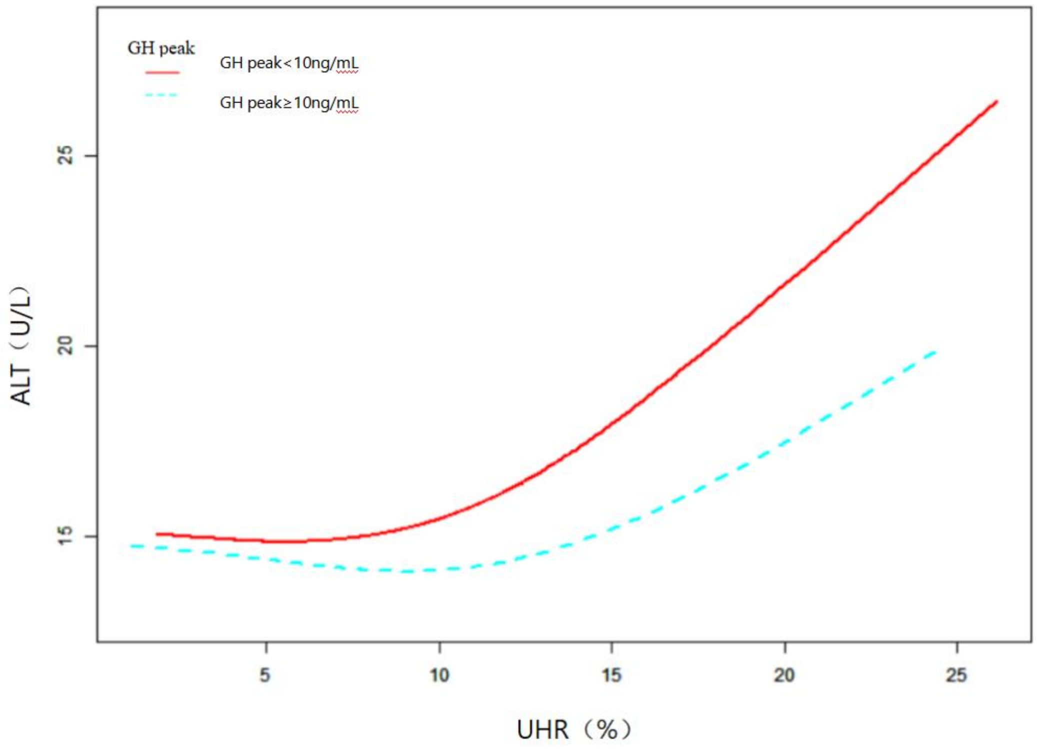

Figure S2: *Smooth curve fitting for the relationship between UHR and ALT stratified by GH peak groups.*

Adjustment variables: sex, age, puberty stage, BMI, SBP, DBP. BMI: body mass index; SBP: systolic blood pressure; DBP: diastolic blood pressure; GH: peak: growth hormone peak; UHR: uric acid to high density lipoprotein cholesterol ratio. p<0.05 was considered to be statistically significant.

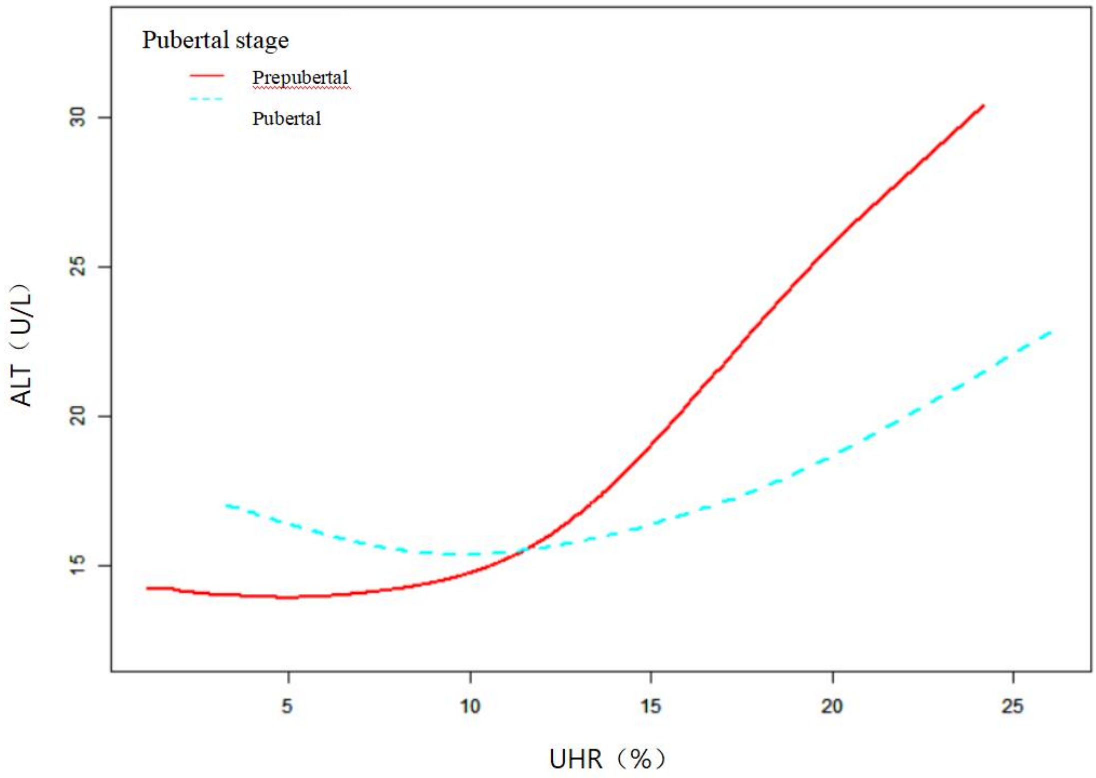

Figure S3: *Smooth curve fitting for the relationship between UHR and ALT stratified by pubertal stage.*

Adjustment variables: sex, age, BMI, SBP, DBP, and GH peak. BMI: body mass index; SBP: systolic blood pressure; DBP: diastolic blood pressure; GH: peak: growth hormone peak; UHR: uric acid to high density lipoprotein cholesterol ratio. p<0.05 was considered to be statistically significant.
